# Computational screening of *Sophora flavescens* phytochemicals as potential aromatase inhibitors: Molecular docking, ADMET, Network pharmacology, Molecular dynamics simulation, and DFT studies

**DOI:** 10.1016/j.pscia.2026.100108

**Published:** 2026-01-20

**Authors:** Laiba Rashid, Jarin Tasnim, Md Mehedi Hasan, Hammad Ismail, Khan Rajib Hossain, Sarnop Sarker, Ayesha Younas, Muhammad Mujtaba, Mohammad Taufiq Alam

**Affiliations:** a School of Chemistry, University of the Punjab, Lahore, Punjab, 54590, Pakistan; b Department of Applied Chemistry and Chemical Engineering, University of Rajshahi, Rajshahi, 6205, Bangladesh; c Department of Biochemistry and Biotechnology, University of Gujrat, Punjab, 50700, Pakistan; d Department of Natural Science, BGMEA University of Fashion and Technology, Dhaka, 1230, Bangladesh; e Materials Engineering, University of Chinese Academy of Sciences, Beijing, 100040, China

**Keywords:** Aromatase (CYP19A1), Molecular docking, ADMET analysis, Network pharmacology, Molecular dynamic simulations

## Abstract

Breast cancer is one of the most prominent types of cancers and a leading cause of fatality in women throughout the world. It also contributes to abnormal estrogen production. The enzyme aromatase (CYP19A1), which catalyzes estrogen biosynthesis, regulates hormone-dependent breast cancer. Therefore, it is used as a therapeutic target in breast cancer therapy. However, long-term usage of FDA-approved aromatase inhibitors, i.e., Letrozole and Anastrozole, in women demonstrates several adverse effects. Thus, the study was conducted to identify safer, natural alternatives using an integrated computational method. In the initial screening, more than 70 phytochemicals from the *Sophora flavescens* plant were docked against aromatase (PDB ID: 3EQM) using PyRx. Though Artocarpin SF02 showed the highest binding affinity among these phytochemicals (−10.1 kcal/mol), it had the highest toxicity to the immune system and the lowest LD50 value compared to SF03. So SF04 Vogelin E, having a binding affinity of (−9.3 kcal/mol), followed by its good pharmacokinetic & toxicity profile. Network pharmacology revealed that it interacts with targets of interest, including CYP19A1, ESR1, and EGFR, which regulate estrogen biosynthesis, as well as PI3K-Akt and MAPK signaling pathways, in breast cancer development. The stability of the aromatase-Vogelin E complex was confirmed by performing MD simulations with GROMACS 2022.4 using the CHARMM36 force field and TIP3P water model, which predicted stable complex formation over the 100 ns trajectory. To further validate the chemical stability and electronic reactivity of the selected compound, DFT calculations were performed using Gaussian 09 with the B3LYP/6-31G basis set. These findings suggest that a flavonoid-based derivative, Vogelin E, derived from the *Sophora flavescens* plant may be a highly effective, safe, and natural aromatase inhibitor. This research offers a good prospect for in vitro and in vivo research to develop alternative therapies to be used in hormone-dependent breast cancer.

## Highlights

• Vogelin E was discovered as a novel natural aromatase inhibitor.
• The stability of the complex was validated through 100 ns molecular dynamics simulations.
• Compared to FDA-approved drugs, it exhibits low toxicity and superior pharmacokinetic properties.
• A safe, flavonoid-based candidate drug for the treatment of breast cancer is proposed.

## Graphical abstract

**Figure:**
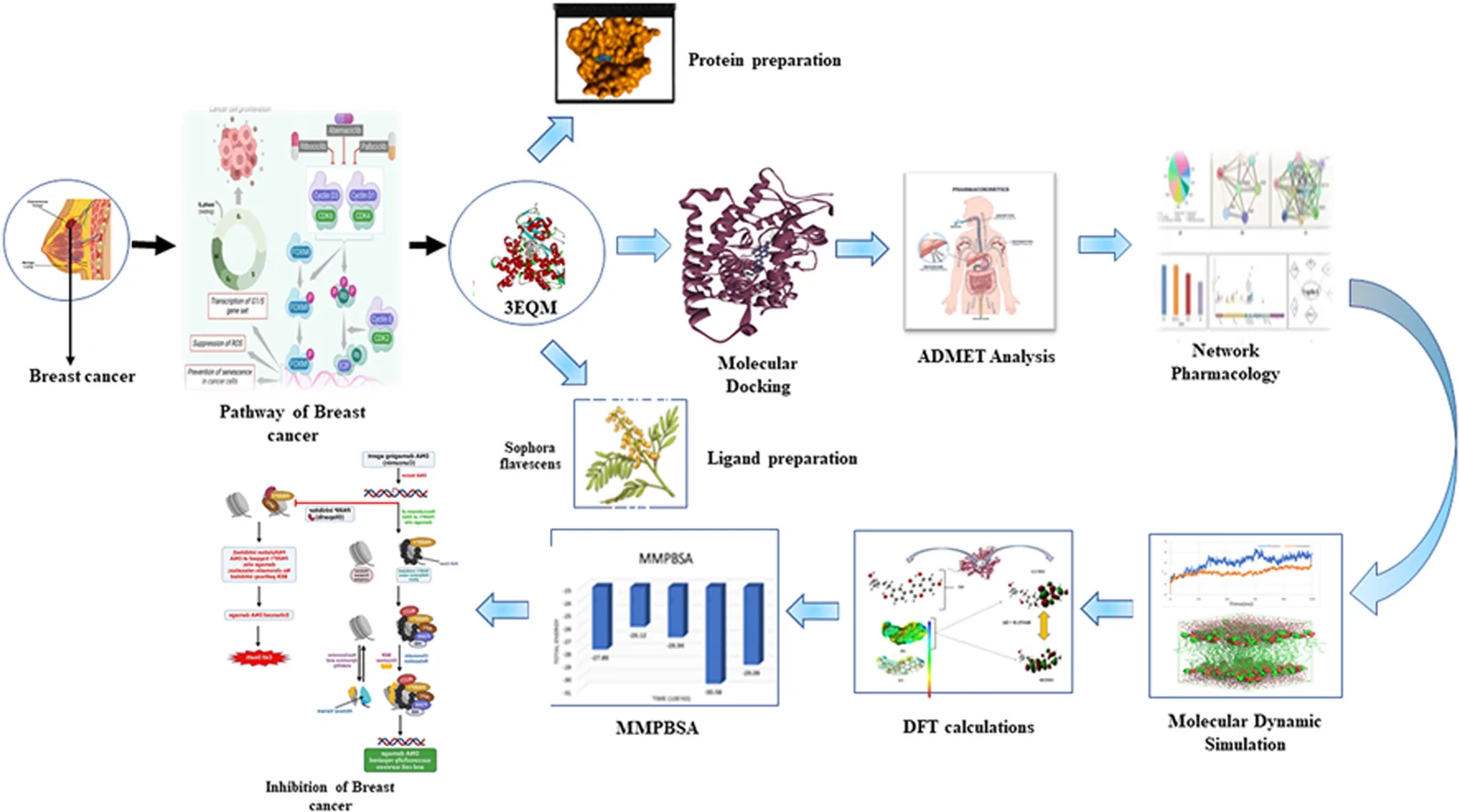

**Table:** Abbreviation list:

ADMET	Absorption, Distribution, Metabolism, Excretion, and Toxicity
BBB	Blood-Brain Barrier
DFT	Density Functional Theory
DILI	Drug-induced liver injury
FDA	Food and Drug Administration
HOMO	Highest Occupied Molecular Orbital
LUMO	Lowest Unoccupied Molecular Orbital
MDs	Molecular dynamics simulations
MMPBSA	Molecular Mechanics Poisson-Boltzmann Surface Area
PDB	Protein Data Bank
QED	Quantitative Estimate of Drug
RG	Radius of Gyration
RMSD	Root Mean Square Deviation
RMSF	Root Mean Square Fluctuation
SASA	Solvent Accessible Surface Area)
TPSA	Total polar surface area
WHO	World Health Organization

## 1 Introduction

Breast cancer is one of the most frequently diagnosed neoplastic diseases among women worldwide. One of its most common types is hormone-dependent breast cancer, which occurs due to aberrant production of estrogen. It is rising as a significant cause of mortality in women throughout the world. In a study of 2022 by the WHO, about 2.1 million women are affected by breast cancer every year [1]. Aromatase is a key enzyme responsible for estrogen biosynthesis and is present in breast tissue [2]. It converts androgens into estrogen, and it is the most common in postmenopausal women, where estrogen production occurs primarily in peripheral tissues rather than the ovaries [3]. Many types of breast cancers rely on estrogen production to propagate. So, aromatase inhibitors are very commonly used in conventional breast cancer therapy. There are FDA-approved inhibitors, such as Letrozole and Anastrozole, known for their ability to inhibit aromatase; however, due to their adverse side effects, including musculoskeletal pains, fatigue, and bone thinning, among others [4]. These risk factors increase the risk of broken bones and decrease patients' overall lifetime quality of life. Therefore, there is a need for safer inhibitors for the hormone-based breast cancer therapy. This study provides an in silico platform to screen more than 70 natural flavonoids from a traditional herbal medicine, *Sophora flavescens*, for CYP19A1 inhibitory activities using molecular docking, ADMET prediction, network pharmacology, molecular dynamics simulations, and DFT calculations, and identifies new safe leads as alternatives to the existing aromatase (CYP19A1)-inhibiting drugs. Aromatase is a key enzyme in estrogen biosynthesis; however, current inhibitors (Letrozole and Anastrozole) have many side effects. Through the discovery of Vogelin E as a naturally promising inhibitor that has good binding and pharmacokinetic properties, this work is committed to providing a safer drug for hormone-dependent breast cancer.

In modern times, network pharmacology and in silico drug designing have gained popularity for the development of potential therapeutics for various diseases before clinical trials. In this study, we employed several computational methods, including molecular docking, ADMET analysis, Network pharmacology, molecular dynamics simulations, and DFT calculations, to identify natural aromatase inhibitors for hormone-based breast cancer therapy. Natural compounds have long been at the center of drug discovery. In recent years, flavonoids have attracted significant attention for their multi-target pharmacological properties, including antioxidant, anti-inflammatory, and anticancer activities. Their favorable ADMET profile**s** further enhance their potential as promising candidates for the development of safer and more effective therapeutic agents [[5], [6], [7]]. For our research, approximately 70 natural compounds from *Sophora flavescens* were obtained from Dr. Duke's phytochemical database. These compounds were assessed against aromatase (PDB ID: 3EQM) to identify the best inhibitors. Molecular docking, ADMET analysis, and further computational procedures were performed to determine the most effective aromatase inhibitors based on their binding affinities, with FDA-approved inhibitors used as standards for comparison. Among them, Kurarinone, along with its structurally similar top six analogs, is shown as the most effective inhibitor, based on its binding affinities and safety profiles. Out of the top six inhibitors, Vogelin E showed the best ADMET profile and drug-likeness characteristics. For further confirmation, network pharmacology was employed to identify distinct signaling pathways involved in regulating aromatase in breast cancer. The 100 ns molecular dynamics simulation analysis was performed to validate the stability of the Vogelin E Aromatase complex. To analyze the electronic properties, a DFT calculation of the selected compound was carried out using *Gaussian.* This investigation suggests that of the Desoxy-analogs, Vogelin E is clearly the most potent one for hormone-based breast cancer treatment. Among Kurarinone analogs obtained from *Sophora flavescens* on s.o., we have found promising clinical data indicating greater in vivo safety and a more favorable side-effect profile compared to several currently used drugs, which support their consideration as interesting candidates for further pre-clinical testing. Vogelin E (SF04) was identified as the most potent inhibitor, revealing a novel lead compound with relevant therapeutic potential for the treatment of hormone-dependent breast cancer. It should be noted that predictive in silico data can only furnish a preliminary indication and do not provide evidence of clinical efficacy or safety. Therefore, the comparison of our compounds with the established FDA-approved aromatase inhibitors in the current study is based solely on docking scores and predicted ADMET profiles, which must be further confirmed experimentally. Although in silico studies have reported natural or synthetic AIs, to date, no integrated study has systematically profiled the wide range of flavonoids in *Sophora flavescens* against CYP19A1. Prior work is limited to single chemicals, and combined screening across docking, pharmacokinetics, and network pharmacology still lags. This study was distinct because it incorporated six TCM models into a docking simulation to treat CYP19A1 DNA. This study adds novelty by screening over 70 phytochemicals, narrowing down several flavonoid subclasses, and selecting Vogelin E (SF04) as a new potential aromatase inhibitor based on multi-computational analyses. This combination therapy strategy offers novel mechanistic information for *Sophora flavescens* flavonoids and their application in hormone-dependent breast cancer treatment.

## 2 Materials and methods

### 2.1 Preparation of protein and ligands

The molecular docking-integrated ADMET and toxicity profiling, network pharmacology, combined with MD simulations and DFT approaches, establish a robust multi-tiered computational workflow to enhance the rigour of discovery and validation of novel AIs. The three-dimensional structure of aromatase (CYP19A1; PDB ID: 3EQM) was available in the Protein Data Bank. The missing residues were filled in using Modeller version 10.7 to produce a full protein structure suitable for computer analysis. Using data from Dr Duke's Phytochemical and Ethnobotanical Database, the PubChem database produced ligand structures for more than 70 phytochemicals from *Sophora flavescens*. All water molecules and heteroatoms present in the crystal were removed before docking. The protein structure was prepared by adding polar hydrogens using Discovery Studio Visualiser. In order to do molecular docking analysis later, ligands were energy-minimized and converted into PDBQT format using Open Babel. [8]. Their IDs were taken from the PubChem database [9]. The 3D structure of aromatase (PDB ID: 3EQM) was sourced from the Protein Data Bank (PDB). This protein includes missing residues, which were added by using Modeller 10.7. The number of amino acids, ranging from 1 to 44 and from 496 to 500, was added to avoid any interruption for further computational analysis. The best docked structure was confirmed by the DOPE energy profile, which reached a stereochemically acceptable conformation. Structural examination was performed using a pocket detection implemented in the Discovery Studio Visualizer, with no steric hindrance and distorted angles being observed. Ramachandran plot, which showed 92.4% residues in the most favoured region with no outliers (Fig. 1), and a further stereochemical check was performed to validate the model's stereochemistry using ProCheck analysis. The validity of the reconstructed regions was confirmed using a variety of structural analysis methods. The lowest-energy model obtained after energy minimization was selected among the decoys using Modeller's DOPE (Discrete Optimized Protein Energy) scoring function. The modelled loops were also checked against homologous CYP19A1 structures to determine whether they are compatible with the expected patterns of secondary structure. Only the models that passed all quality checks were moved forward for docking. Before the molecular docking procedure, all heteroatoms and water molecules were removed from the protein using Discovery Studio Visualizer, and hydrogen bonds were incorporated into the protein. For the ligands, open Babel was used to compress them into a single file for export to PyRx.

**Fig. 1:**
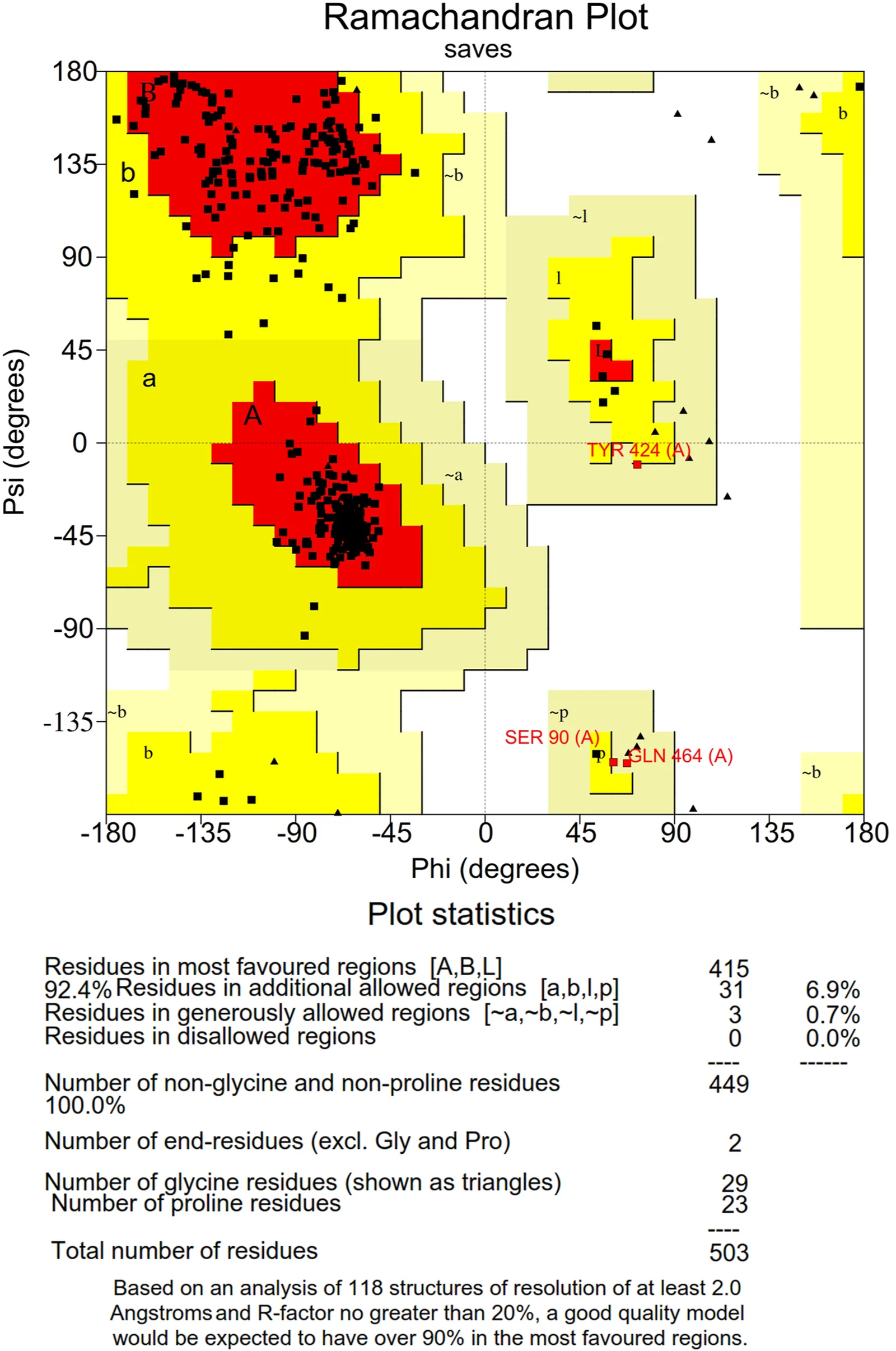
Ramachandran plot obtained from PROCHECK for the validation of added missing residues.

### 2.2 Molecular docking

Molecular docking was performed using PyRx, and AutoDock Vina, with all parameters for precise docking set within the software [10]. All the ligands and proteins were uploaded into the software. The protein was converted to pdbqt format by treating it as a macromolecule. All ligand energies were minimized and converted to pdbqt format before docking. The grid box was positioned at the active site (heme bound by CastP), with coordinates of x = 85.370, y = 50.330, z = 41.570, and its centers at x = 28, y = 26, z = 26, to determine the accurate placement of ligands. PyMOL was used to export the docked complexes, and Discovery Studio Visualizer was used to visualize the docking interactions. The docking scores of top-scoring compounds are listed in Table 2, along with their corresponding interactions. The ADME analysis was performed using ADMETlab 3.0 and SwissADME, shown in Table 3. The toxicity of all the mentioned ligands was evaluated using Protox 3.0, as shown in Table 4 [[11], [12], [13]].

**Table 1:** *Sophora flavescens* flavonoids (SF01-SF06) with their Chemical names, PubChem IDs, and structures.

Compounds	Chemical Name/CID no.	SMILES	Structures
**SF01**	11982640 Kurarinone	CC(=CC[C@H](CC1=C2C(=C(C=C1O)OC)C(=O)C[C@H](O2)C3=C(C=C(C=C3)O)O)C(=C)C)C	
**SF02**	5458461 Artocarpin	CC(C)/C=C/C1=C(C=C2C(=C1O)C(=O)C(=C(O2)C3=C(C=C(C=C3)O)O)CC=C(C)C)OC	
**SF03**	11728770 Euglobal IIC	CC(C)CC(=O)C1=C(C(=C2C(=C1O)C[C@H]3C[C@@H](C=C[C@]3(O2)C)C(C)C)C=O)O	
**SF04**	10904337 Vogelin E	C/C(=C\CC1=C(C=CC(=C1)C2=COC3=CC(=CC(=C3C2=O)O)O)O)/CO	
**SF05**	72936 Sophora flavanone G	CC(=CC[C@H](CC1=C2C(=C(C=C1O)O)C(=O)C[C@H](O2)C3=C(C=C(C=C3)O)O)C(=C)C)C	
**SF06**	5281804 Prunetin	COC1=CC(=C2C(=C1)OC=C(C2=O)C3=CC=C(C=C3)O)O

**Table 2:** (SF01-SF06) with their binding energies and interactions, including standard inhibitors.

Compounds	Binding Energy Kcal/mol	Hydrogen Bond Interactions	Van der Waal residues & others
**SF01**	−9.3	ARG145, ALA 306	ARG115, ILE 132,ILE133,TRP141, PHE148,LEU152,TRP224,GLU302, MET303,ASP309,THR310,VAL370, ARG435,GLY436,CYS437,ALA438, GLY439,SER478
**SF02**	−10.1	ARG115,MET 374, ALA 438	ILE132,PHE134,ARG145,PHE148, LEU152,PHE221,TRP224,MET303, ALA306,ASP309,THR310,VAL369, VAL370,LEU372,VAL373,GLY436, CYS437,GLY439,LEU477,SER478
**SF03**	−9.4	ARG115,CYS 437,ALA438, GLY439	ILE132,ILE133,ARG145,PHE148, LEU152,GLU302,MET303,THR310, VAL370,LEU372,VAL373,MET374, PHE430,ARG435,GLY436,LEU477, SER478
**SF04**	−9.3	ARG115,SER 199,MET303, ALA307	ILE132,ILE133,PHE148,LEU152, PHE203,TRP224,GLU302,ALA306, MET311,VAL373,MET374,CYS437, ALA438,GLY439,MET446
**SF05**	−9.1	ARG115, PRO 429	ILE133,PHE134,PHE221,TRP224, ALA306,ASP309,THR310,VAL370, LEU372,VAL3733,MET374,PHE430, CYS437,ALA438,ALA443,LEU477
**SF06**	−8.6	ARG192,PRO 308,ASP309	GLN218,PHE221,ASP222,GLN225, THR310,SER312,VAL313,ILE474, HIS480,GLU483,THR484
**LETROZOLE (standard)**	−8.1	ARG435,GLY436	ARG115,ILE132,ILE133,PHE134, TRP141,ARG145,PHE148,LEU152, PHE203,TRP224,MET303,ALA306, VAL370,CYS437,ALA438,GLY439, LEU477
**ANASTROZOLE (standard)**	−7.4	ILE132,ALA306, GLY436	ARG115,ILE133,TRP141,PHE148, LEU152,TRP224,GLU302,MET303, ASP309,THR310,ARG435,CYS437

**Table 3:** Physiochemical, Medicinal, Lipophilicity, and Pharmacokinetic properties of selected flavonoids (SF01-SF06).

Compounds	SF01	SF02	SF03	SF04	SF05	SF06
**Physiochemical Properties**						
Molecular weight	438.51	436.5	386.48	354.35	424.49	284.26
Volume	461.596	458.959	408.827	357.82	444.3	282.482
Density	0.949	0.95	0.945	0.99	0.955	1.006
nRot	7	6	5	4	6	2
nHA	6	6	5	6	6	5
nHD	3	3	2	4	4	2
TPSA	96.22	100.13	83.83	111.13	107.22	79.9
**Medicinal Properties**						
QED	0.486	0.425	0.436	0.536	0.468	0.756
Fsp3	0.346	0.269	0.565	0.15	0.32	0.062
SAscore	4.55	4.43	4.65	3.45	4.44	2.96
Lipinski Rule	Yes	Yes	Yes	Yes	Yes	Yes
Pfizer Rule	Yes	Yes	Yes	Yes	Yes	Yes
Golden Triangle Rule	Yes	Yes	Yes	Yes	Yes	Yes
Bioavailability	0.55	0.55	0.55	0.55	0.55	0.55
PAINS	0	0	0	0	0	0
**Lipophilicity**						
iLOGP	3.08	4.14	3.3	2.47	3.14	2.5
XLOGP3	5.55	5.72	5.38	3.34	5.78	2.99
WLOGP	5.29	5.65	4.68	3.06	4.98	2.88
MLOGP	2.48	2.3	2.37	0.82	2.28	0.77
SILICOS-IT	5.5	6.08	6.08	3.6	4.95	3.03
Consensus LOG *Po/w*	4.38	4.78	4.78	2.66	4.23	2.43
**Pharmacokinetics Properties**						
GI absorption	High	High	High	High	High	High
BBB permanent	No	No	No	No	No	No
P-gp substrate	Yes	No	No	No	No	No
CYP1A2	No	No	No	Yes	No	Yes
CYP2C19	No	Yes	No	No	No	No
CYP2C9	Yes	No	Yes	No	Yes	No
CYP2D6	No	No	No	Yes	No	Yes
CYP3A4	Yes	No	Yes	No	Yes	Yes
Log Kp cm/s	−5.03	−4.9	−4.84	−6.09	−4.79	−5.91

**nRot:** No. of rotational bonds, **nHA:** No. of hydrogen bond acceptors, **nHD:** No. of hydrogen bonds donor, **TPSA:** total polar surface area, **QED:** Drug-likeness score, **GI:** Gastrointestinal Absorption, **BBB:** Blood Brain Barrier, **P-gp:** P-glycoprotein, **CYP:** Cytochromes, LogKp: Skin penetration.

**Table 4:** Toxicity parameters of (SF01-SF06) by AMDETlab 3.0 & protox 3.0.

Toxicity profile	SF01	SF02	SF03	SF04	SF05	SF06
Class	4	5	4	5	4	5
LD50 mg/kg cm/s	2000	4000	860	2500	2000	2500
Aquatic Toxicity Rule	0	0	0	1	0	0
Genotoxic/Carcino/Mutagenicity Rule	0	0	0	1	0	0
Acute Toxicity Rules	0	0	0	0	0	0
Non-Biodegradable Rule	1	1	1	1	1	0
DILI	0.577	0.717	0.118	0.92	0.555	0.822
AMES Toxicity	0.631	0.34	0.204	0.454	0.677	0.546
hERG blockers	0.135	0.085	0.082	0.083	0.083	0.161
FDAMDD	0.858	0.864	0.737	0.809	0.894	0.646
Eye corrosion	0.01	0.001	0.022	0	0.006	0.069
Eye irritation	0.964	0.754	0.929	0.944	0.978	0.977
Environmental Toxicity						
BCF	1.813	1.666	2.021	1.305	1.849	1.23
IGC50	4.835	5.071	4.808	4.019	4.922	3.862
LC50DM	6.45	6.526	6.401	5.26	6.463	4.554
LC50FM	5.848	0.083	5.925	4.734	5.843	4.155

**hERG:** human ether-a-go-go related Gene [31], **DILI:** Drug-induced liver injury [32], **FDAMDD:** FDA maximum daily dose [33], **BCF:** Bio-concentration factor.

### 2.3 Network pharmacology

SwissTargetPrediction, the STRING database, and g: Profiler were used to study network pharmacology [[14], [15], [16]]. SwissTargetPrediction was used to study the functional classification of different targets of a molecule, using target prediction probabilities ≥0.1. SwissTargetPrediction was filtered to retain only targets with a predicted probability >0.10 to reduce noise from low-confidence predictions. Duplicates and ES-2904 targets not found in human data were filtered out. The protein-protein interaction network was also generated for STRING analysis at a high confidence level (0.70), with a score of evidence, and comprised experimental, co-expression, and curated database evidence. Interactions with confidence below this threshold (low confidence) were omitted to eliminate weak or computationally uncertain associations. Protein-protein interactions were visualized using the STRING database, with a minimum confidence score of 0.4 to ensure complete and reliable network coverage. A higher confidence score was not used, as it can result in the removal of biologically meaningful interactions and the loss of essential nodes in the networks. Gene ontology and pathway topology (GO & KEGG) were investigated using g: Profiler with the integrated g: SCS (Set Counts and Sizes) correction method, where pathways were considered significant if FDR <0.05.

### 2.4 Molecular dynamics simulations

MD studies were performed both for the apo protein (3EQM) and its complex with the ligand SF04 (Vogelin E) using GROMACS 2022.4 [17]. The simulation trajectory was 100 ns, at 300K, using the CHARMM36 force field and the TIP3P water model. The PDB model was employed to develop the protein topologies, and the ligand was extracted from the docked complex. The remaining charges were adjusted manually, and both the protein and ligand topology files were merged. The atoms of the ligand were incorporated into the complex topology file while other parameters for the system's ligand were updated. Throughout the procedure, the configurations of protein 3EQM and the complex were sampled every 100ns. Essential parameters, such as RMSD, RMSF, RG, and SASA, were determined from the simulation to evaluate the stability of the complex. The graphs were drawn using Microsoft Excel.

### 2.5 Density Functional Theory (DFT)

DFT analysis was performed using Gaussian 9.0 with the 6-31G basis set at the B3LYP level [18,19]. This level for DFT calculation and geometry optimization was selected due to its validation in computational drug designing. While GaussView 6.0 was utilized for drawing the structures and figures [20].

## 3 Results and discussion

### 3.1 Molecular docking analysis

Molecular docking of *Sophora flavescens-*derived compounds was performed against aromatase to assess their potential inhibitory capacity. Based on structural similarity, Kurarinone and related flavonoids were docked to assess their docking scores and drug-likeness. Among which SF01 (Kurarinone) showed the best binding affinity score. Table 1 shows the investigated compounds bound to aromatase (PDB ID: 3EQM), including their chemical names, SMILES, and structures.

These six compounds (SF01-SF06) were analyzed for their inhibitory activity against aromatase in breast cancer. All the compounds were docked into 3EQM to assess their binding energies in the aromatase active. For comparative validation, FDA-approved inhibitors Letrozole and Anastrozole were used as standards. Their docking scores, along with the interacting residues, are shown in Table 2. The spatial interaction characteristics of proteins and small-molecule ligands within their binding sites (Fig. 2). In the structure, the α-helix is red, the β-sheet is cyan, and the ring structure is green, clearly showing the ligand-binding environment in the overall conformation. The ligands are shown in different colors, embedding themselves in the binding channel and forming stable interactions through hydrophobic and multi-site interactions. The ligand-occupancy mode and its influence on local conformation provide support for subsequent structural analysis and mechanism elucidation. Redocking validation showed RMSD <2 Å of the native vs. the Re-docked pose, indicating that the docked poses were reliable. For comparative validation, FDA-approved inhibitors Letrozole and Anastrozole were used as standards. Their docking scores, along with the interacting residues, are shown in Table 2.

**Fig. 2:**
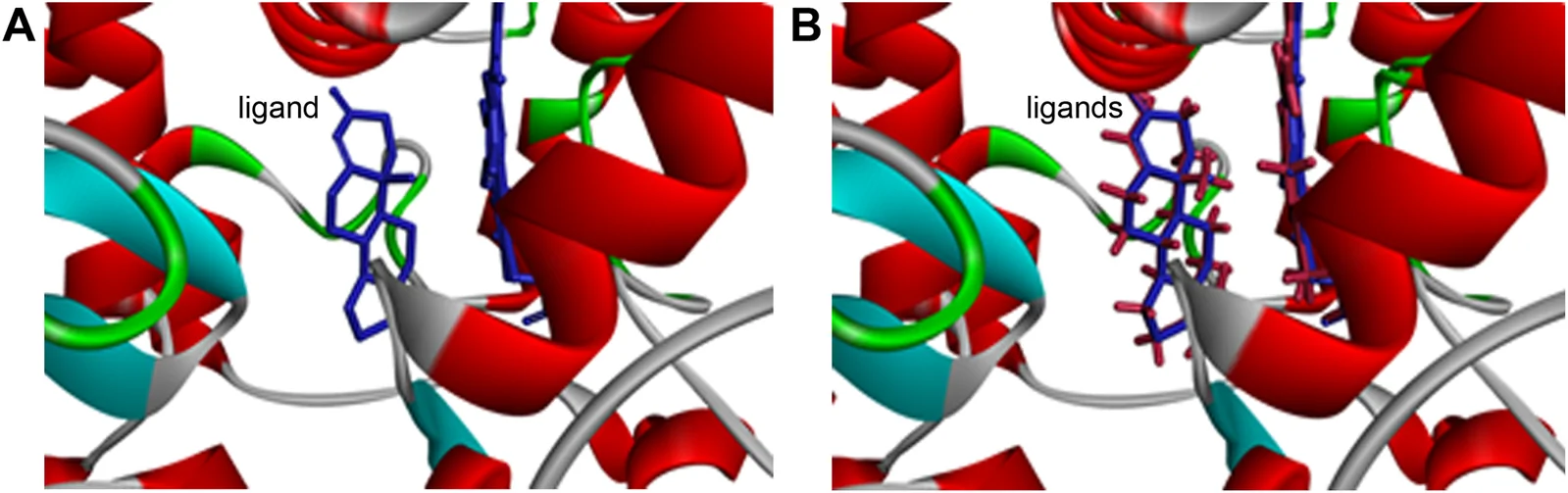
(A) Shows only one ligand (blue) interacting with the protein binding site, surrounded by red α-helices, cyan β-sheets, and green loops, and (B) Re-docked Complex superimposed on the crystallized complex, which shows two ligands binding simultaneously, with the second ligand appearing in purple-red, further occupying the binding channel. (For interpretation of the references to color in this figure legend, the reader is referred to the Web version of this article.)

Natural flavonoids have higher binding affinities than the FDA-approved aromatase inhibitors, i.e., Letrozole and Anastrozole. The 3D and 2D interaction analysis of SF01-SF04 with 3EQM, in contrast to standard inhibitors, is shown in Fig. 3, Fig. 4. The three-dimensional docking poses show how the ligands fit into the active binding pocket of aromatase. The two-dimensional interaction maps show how the intermolecular forces that keep the ligands stable work in more detail. The docked complexes are mostly held together by hydrogen bonds, van der Waals interactions, π-π stacking, π-alkyl, and π-sulphur interactions. van der Waals interactions are shown in green, hydrogen bonds in light green, π-π-sulphur interactions in yellow, π-alkyl interactions in purple, and π-π stacking interactions in pink in the 2D interaction diagrams. These patterns of interaction all work together to make the selected phytochemicals bind in a way that is stable and works well in the aromatase active site (Fig. 3).

**Fig. 3:**
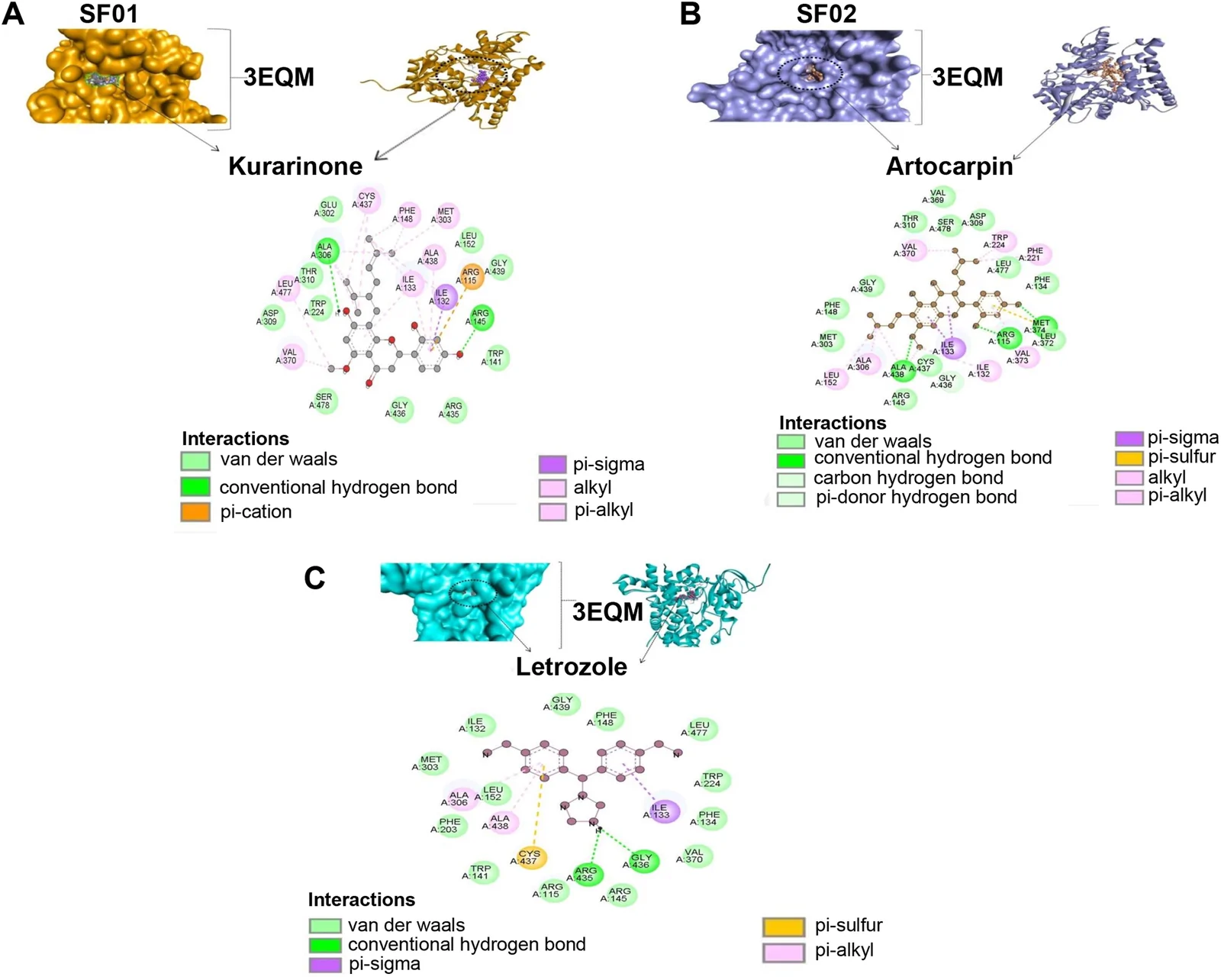
The diagram illustrates the molecular docking interactions between compounds Kurarinone, Artocarpin, and Letrozole and the target proteins SF01, SF02, and 3EQM. (A) Shows the binding mode of Kurarinone with SF01, (B) shows the binding mode of Artocarpin with SF02, and (C) shows the complex structure of Letrozole with 3EQM. The three-dimensional structure illustrates the binding of ligands at the protein active sites. Three-dimensional (3D) and two-dimensional (2D) molecular docking interactions of chosen *Sophora flavescens* phytochemicals and standard inhibitors with aromatase (PDB ID: 3EQM).

**Fig. 4:**
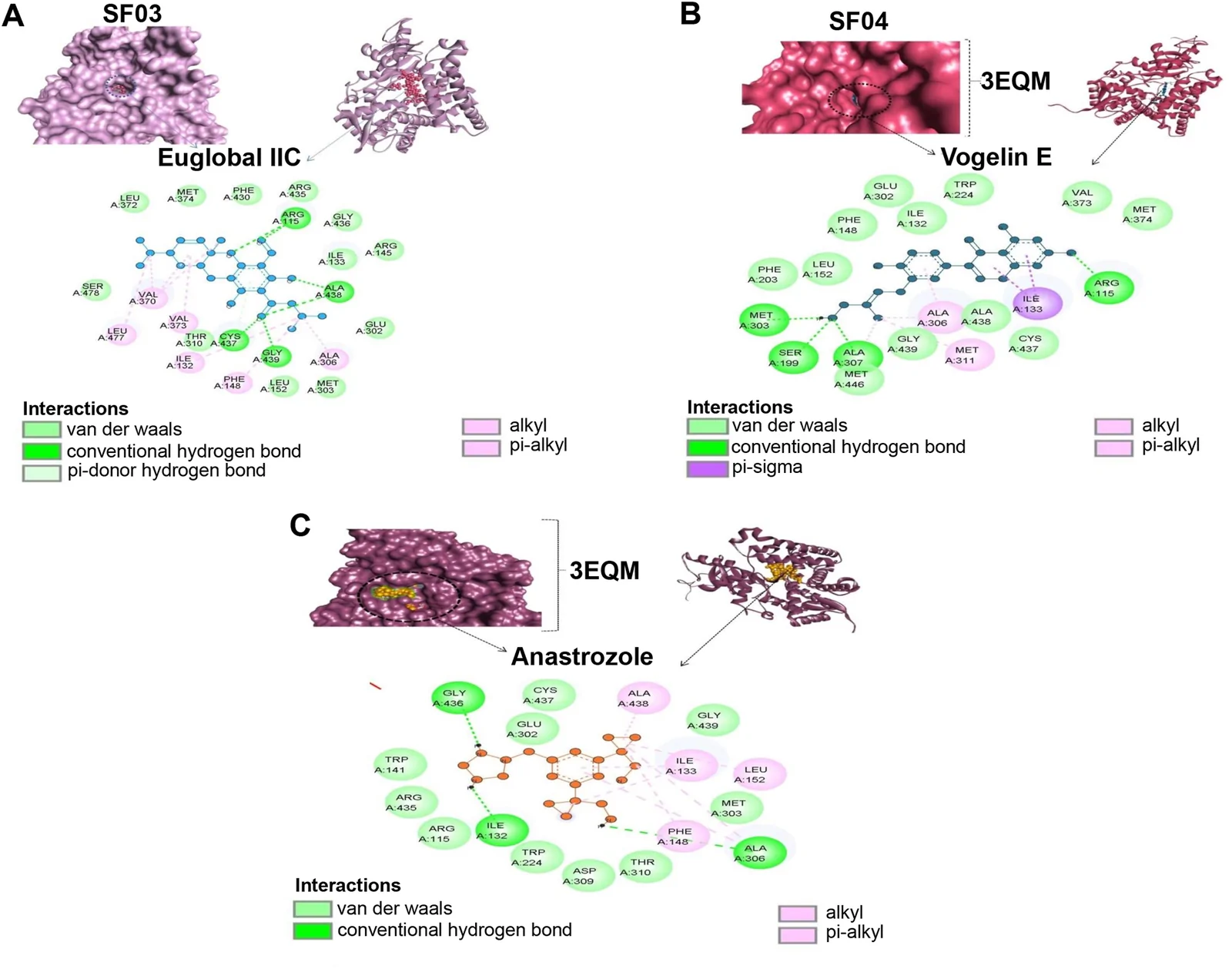
3D and 2D docking visualization of SF03 & SF04 in comparison with Anastrozole. (A) Shows the molecular docking results of Euglobal IIC and SF03, (B) illustrates the binding mode of Vogelin E and SF04, which primarily achieves binding through van der Waals forces, hydrogen bonds, and π-alkyl interactions, thereby demonstrating high affinity, and (C) shows the binding of the standard drug Anastrozole with 3EQM.

The docking analysis indicated that SF03 and SF04 bind to the active core region of aromatase in a way that is similar to how the standard inhibitor anastrozole binds. SF03 forms several hydrogen bonds and hydrophobic interactions in the catalytic pocket, which help the ligand stay stable. SF04 also shows good binding thanks to a mix of van der Waals forces and hydrogen bonding interactions, which leads to a stable protein-ligand complex. The interaction pattern of anastrozole serves as a benchmark for comparative analysis, emphasizing the analogous binding orientation and interaction profile of the chosen phytochemicals within the aromatase's active site (Fig. 4).

### 3.2 ADMET analysis

A systematic ADMET profile is presented that leverages rule-of-five filtering, QED scoring, lipophilicity computations, cytochrome P450 interaction predictions, and DILI Risk modeling for a comprehensive, in-depth pharmacokinetic and safety evaluation.

#### 3.2.1 Physicochemical properties

The fundamental criterion for compounds to be a suitable drug candidate is evaluated by Lipinski's Rule of Five. Any violation of this rule leads to poor absorption and permeability [21]. The significant characteristics of this rule are molecular weight ≤500, hydrogen bond donors ≤5, hydrogen bond acceptors ≤10, and logP ≤5 to signify lipophilicity. The given attributes are approximately associated with a 90% oral bioavailability of the drug [22]. The molecular weights of SF01, SF02, SF03, SF04, SF05, and SF06 are 438.51, 436.5, 386.48, 354.35, 424.49, and 284.26, respectively. The number of rotatable bonds for SF02 and SF05 is 2, while for SF01, SF03, SF04, and SF06 is 7, 5, 4, and 2, respectively. The number of hydrogen bond acceptors for all the investigated natural compounds is in the range of 5–6, and the number of hydrogen bond donors is in the range of 2–4. For that, the physicochemical properties of SF01-SF06 need to be in the required range. The total polar surface area (TPSA) for all the studied compounds is within the range of 79–111.

#### 3.2.2 Medicinal properties

The QED scores for SF01, SF02, SF03, SF04, SF05, and SF06 are in the range of 0.4–0.7, which is between a moderate and high range. SF01, SF02, SF03, and SF05 are weak for drug likeness; SF05 is mild, and SF06 is the strongest candidate for a drug. For a molecule to be attractive, the QED score should be greater than 0.67; for an unattractive molecule, it is between 0.49 and 0.67, and for too complex, it is less than 0.34, because drugs with a high score are better for absorption [23,24]. Fsp^3^ is a proportion of hybridized sp^3^ carbon atoms, which indicates the saturation of the molecule and the spatial complex structure. All compounds are accepted according to the Lipinski Rule, the Pfizer Rule, and the Golden Triangle Rule. The Pfizer rule exhibits two characteristics: a TPSA of less than 75 and a logP greater than 3. After examining 245 chemicals, Pfizer found that compounds with TPSA greater than 75 and logP less than 3 are non-toxic. The Golden Triangle Rule utilizes three visualization tools, including computational data, in vitro clearance, and in vitro permeability, for drug design. All the compounds fulfill this criterion. PAINS demonstrate unrestricted behavior that can lead to false positive results due to non-covalent interactions, and the exact mechanism is unknown. For that, there are no alerts for all the investigated compounds (SF01-SF06).

#### 3.2.3 Lipophilicity

Lipophilicity characteristics are essential for evaluating the drug-likeness of compounds using computational methods. iLOGP, XLOGP3, WLOGP, MLOGP, SILICOS-IT collectively predict consensus LOGP values. Consensus LOG*Po/w* is actually the logarithm of the partition coefficient between n-octanol and water [25]. The optimal range for consensus LOGP is considered to be < 5, and almost all the studied compounds (SF01-SF06) exhibit values within this range, maintaining a balance between hydrophilicity and lipophilicity. It also leads to good membrane permeability.

#### 3.2.4 Pharmacokinetic properties

The absorption, distribution, metabolism, and excretion properties are analyzed for (SF01-SF06) ligands as shown in Table 3. GI absorption, blood-brain barrier (BBB) permeability, Cytochromes enzyme inhibition, and skin penetration values are evaluated for the investigated ligands. High GI absorption for all the studied ligands, and none of them can cross the blood-brain barrier, which is effective for non-CNS drugs, making them good candidates for oral bioavailability, absorption, and permeability, as shown in Table 3. All the compounds are unaffected by P-gp substrates, leading to high levels of absorption, except for SF01 [26]. P-gp substrates are the efflux transporters that limit the accumulation of drugs in the body [27]. SF01 is a P-gp substrate, resulting in poor absorption. Cytochrome inhibitors can alter the metabolism of co-administered drugs, leading to drug-drug interactions [28]. SF04 and SF06 are CYP1A2 inhibitors, while the others are unaffected by it. Mostly are unaffected by the CYP2C19 except SF02, CYP2C9 except SF01, SF03 and SF05, CYP2D6 except SF04 and SF06, CYP3A4 except SF01, SF03, SF05 and SF06. The logKp values for all the compounds fall within the range of −4 to −6 cm/s, indicating moderate to low skin permeability and favoring oral administration.

#### 3.2.5 Toxicity

The higher a compound's toxicity class, the lower its toxicity. SF01, SF03, and SF05 belong to class 4 according to Protox 3.0 toxicological predictions, while the others belong to class 5, indicating the least toxicity. LD50 values are inversely related to a compound's toxicity; the higher the LD50, the lower the toxicity. However, some compounds are highly immunotoxic, even with a high LD50 value, so highly active immunotoxicity may lead to poor absorption. There are no alerts for aquatic, acute, genotoxicity, mutagenicity, or carcinogenicity rules. Drug-induced liver injury values for SF01, SF02, SF03, SF04, SF05, and SF06 are 0.577, 0.717, 0.118, 0.92, 0.555, and 0.822. The fundamental reason for drug failure in clinical trials is drug-induced liver injury (DILI). Therefore, virtual screening of drug candidates is suitable before undergoing clinical trials [29]. SF06 shows a high risk for DILI, while SF01 and SF05 show a moderate risk. SF02, SF03, and SF04 show the lowest risk. The values for hERG blockers range from 0.085 to 0.132, as shown in Table 4. During drug designing, heart problems are the common side effects related to the inhibition of the potassium channel (hERG blockers) [30]. Values less than 0.5 indicate the least cardiac toxicity, while values closer to 1 indicate intense cardiac toxicity. AMES toxicity, FDAMDD, and other factors are shown in Table 4.

#### 3.2.6 Environmental toxicity

All the eco-toxicological factors are mentioned in Table 4. Bioconcentration factors, IGC50, and LC50 values are discussed in the context of environmental toxicity. While considering the possibilities of toxicological effects of drugs and health hazards to humans through the food chain, BCF factors are taken into consideration [34]. While environmental toxicity-related parameters were also correlated with ADMET lab outputs, only a portion is relevant for early drug design. Common to all compounds investigated was a BCF <3, indicating low bioaccumulation potential and thus their potential as small-molecule drug candidates. Other ecotoxicological parameters (IGC50, LC50, BCF; species-specific toxicity) are not directly relevant for pharmacological development and were thus not used to derive compound preference. For completeness, these values are provided only in Table 4 and do not affect the ranking of SF04 for follow-up. *Daphnia magna*, *Tetrahymena pyriformis*, and fathead minnows are primarily used to assess ecosystem and environmental safety, using the 50% inhibitory concentration (IGC50) to calculate the toxicity of aromatic organic compounds [35]. Higher LC50 values represent less toxicity. SF04 performed quite well in ADMET, MD stability, and network pharmacology, as well as in DFT analyses; however, it did not have the best docking score, even though that clearly belongs to SF02 (Artocarpin: −10.1 kcal mol^−1^). SF04 emerged as the lead candidate due to its overall favorable profile, as mentioned above. Docking scores, ADMET predictions, and MD simulations may indicate a compound's potential but are not a substitute for biochemical assays, enzyme inhibition studies, or in vitro and in vivo toxicological investigations. Therefore, SF04 may be a good substitute for existing AIs for further experimental verification rather than a confirmed, more excellent competitive inhibitor. In comparison with previous (computational) aromatase inhibition studies, most of which focus on a single phytochemical compound or synthetic analogs or use more restricted docking-only strategies, the present study offers several improvements. Firstly, to the best of our knowledge, this screening is the most comprehensive study on *S. flavescens* phytoconstituents to date and encompasses over 70 structurally diverse flavonoids. Second, identification of Vogelin E (SF04) as an antiaromatase was not previously reported, and no published work has considered this compound as an antiaromatase using network pharmacology, MD simulations, and DFT analyses of its inhibitory effect on CYP19A1. Third, the inclusion of target-pathway analysis adds a mechanistic underpinning that is lacking in previous studies. Thus, the significance of this report lies not only in the compound screening scale but, more importantly, in a multi-tier validation process that validates SF04 as a high-potential candidate.

#### 3.2.7 Network pharmacology & functional classification

SF04 (Vgelin E) exhibits a moderate to good ADME and toxicity profile, while others show high toxicity to the immune system, and some compounds have low binding affinity. That is why it has been chosen as the best compound for further network pharmacology studies at different levels. In the functional classification, other targets of Vogelin E are identified in the figure, exhibiting a more focused and selective interaction profile, including aromatase (CYP19A1). Such a limited, focused interaction with the CYP450 class highlights its enzymatic regulation rather than its broader impact on other metabolic processes. Ligand-based explorations, coupled with system-level examinations, PPI networks, and pathway enrichment, are incorporated into the method, providing a comprehensive framework that deepens interpretation while maintaining biological relevance. The SwissTargetPrediction output identified 21 human targets with probability ≥0.10. Low-probability relationships (<0.10) were eliminated owing to poor predictive credibility. STRING PPI mapping at high confidence included a cutoff batch file (score ≥0.70), generating a 42-interaction network comprising only experimentally validated or co-expressed associations. Such filtering allows the analysis to focus on biologically relevant associations and ignore algorithmic noise.

#### 3.2.8 Protein-protein interactions & gene ontology

In PPI network interactions, Vogelin E interacts with various proteins, including ESR1, AR, and EGFR, which are involved in the estrogen signaling pathway and PI3K-Akt signaling pathway. The gene CYP19A1 also interacts with other proteins, which directly link with steroid hormone metabolism and biosynthesis in breast cancer. The Vogelin E represents its pharmacological effects through the influence of enzymatic proteins and signaling pathways (Fig. 5A–C). The analysis of gene ontology and KEGG pathway analysis describes the behavior of target genes of Vogelin E related to the signaling pathways involved in breast cancer regulation. Steroid hormone biosynthesis, estrogen signaling, PI3K-Akt, and MAPK signaling pathways were characterized by cell proliferation, survival, and endocrine regulation in breast carcinoma (Fig. 5D and E). These observations suggest that Vogelin E may impact estrogen receptor-based pathways and disrupt proliferative signaling, which could be potentially valuable as a multi-target reagent in breast cancer treatment. GO and KEGG terms were considered significantly enriched when the FDR was <0.05. Based on these considerations, estrogen signaling, PI3K-Akt signaling, MAPK signaling, and steroid hormone biosynthesis were identified as the most enriched categories.

**Fig. 5:**
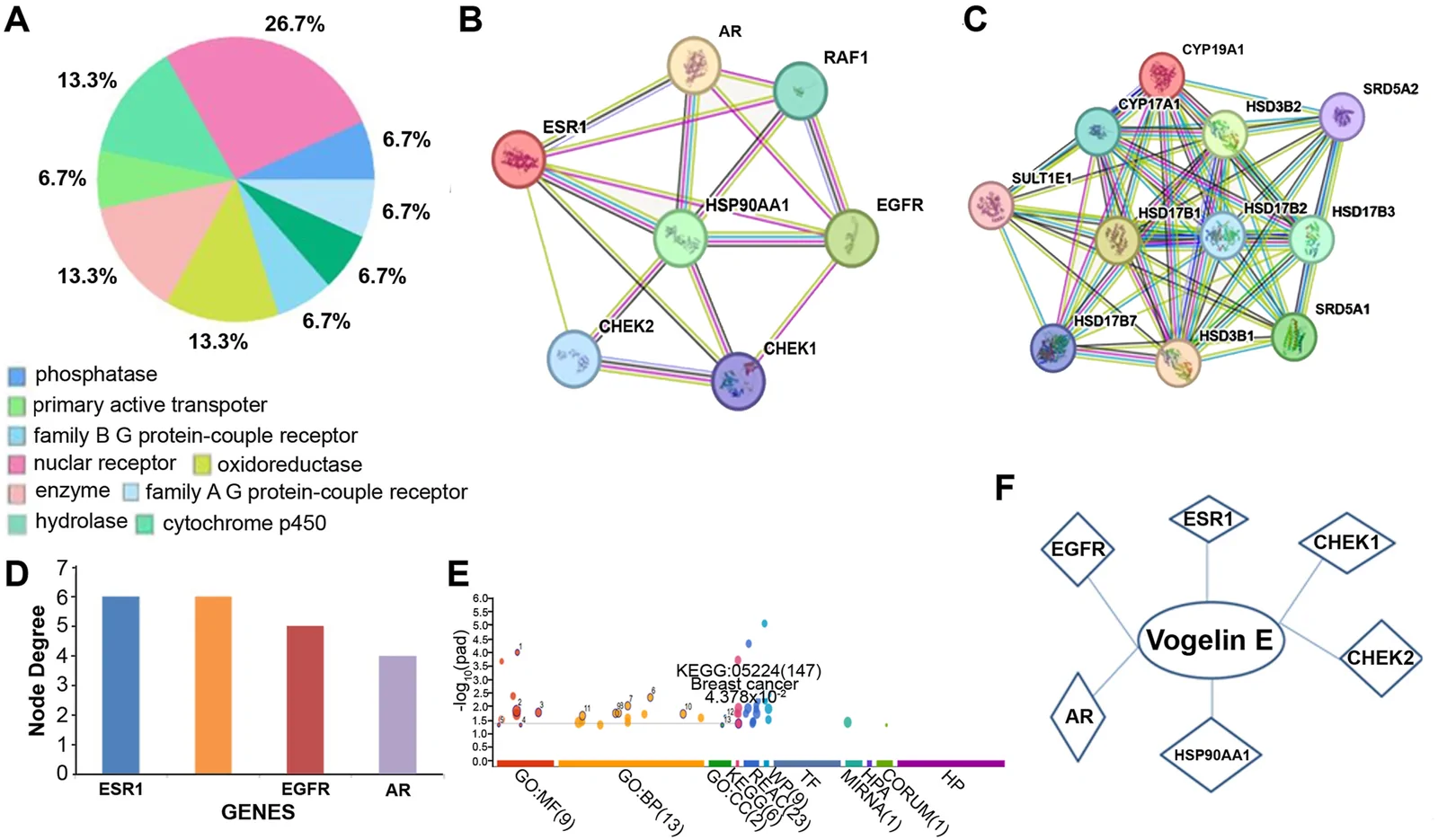
(A) Classification results of Vogelin E's targets, with different colors representing functional types such as phosphatases, nuclear receptors, cytochrome P450, and oxidoreductases, (B) Interaction network (PPI) between potential Vogelin E target proteins, including core nodes such as ESR1, AR, EGFR, and HSP90AA1, (C) Interaction network of steroid metabolism-related proteins, revealing the metabolic pathways that Vogelin E may affect, (D) Key genes ESR1, HSP90AA1, EGFR, and AR were identified through node degree analysis, (E) KEGG enrichment analysis showed that Vogelin E-related targets are closely related to breast cancer and hormone signaling pathways, and (F) Construction of a network model of the interaction relationships between Vogelin E and its main targets. (For interpretation of the references to color in this figure legend, the reader is referred to the Web version of this article.)

#### 3.2.9 Pathway topology, MDs, temperature, pressure, density, and energy

Pathway topology describes how different genes related to breast cancer disease interact with Vogelin E. Based upon the gene ontology results, Vogelin E interacts with ESR1, AR, EGFR, HS90AA1, CHEK1, and CHEK2 by describing estrogen signaling pathway, PI3K-Akt pathway, HF-1 signaling pathway, and cell cycle pathways due to uncontrolled cell cycle progression. MDs are often used to analyze the stability of complexes, based on binding affinity scores and hydrogen-bond interactions. SF02 presented a challenge to the immune system, and SF03 demonstrated the minimum LD50 in the ADMET profile. For further studies between the two, neither was included in the computational analysis, and the one, i.e., SF04 Vogelin E, which possesses the best pharmacokinetic properties and also displays good binding affinity, was selected for subsequent computational studies. Here is the study of SF04 (Vogelin E) with aromatase (PDB ID: 3EQM) based on the best ADMET analysis and binding affinity. The 100 ns duration of the MD simulation is considered sufficiently long to observe significant conformational changes and to assess the long-term stability of the aromatase Vogelin E complex. The complex was then immersed in a cubic box with a minimum 10 Å distance from the edges, using the TIP3P water model. To quench the system, a set of ionic countercharges was added, and 0.15 M NaCl was included to replicate the physiological ion strength. Energy minimization to relieve clashes was performed, followed by NVT and NPT equilibration with restraints on heavy atoms. Temperature and pressure were maintained in the system at 300K and 1 bar, as shown in Fig. 6 for the MD procedure. The fluctuations in temperature and pressure were monitored to ensure the system's stability during MD's trajectory. The system confirms the thermostat-barostat control, which maintains the stability of the ligand-protein complex. The protein density showed a higher trajectory up to 1042–1045 kg/m^3^ compared with the complex, which maintained stability at 1030–1035 kg/m^3^ throughout the 100 ns simulations, indicating a tighter, more compact complex. At the same time, the total energy of the protein fluctuated at −1.32 × 10^6^ kJ/mol, while the complex maintained stability at −1.30 × 10^6^ kJ/mol. The complex displayed slightly lower density and marginally higher energy due to ligand-induced conformational adjustments. A 1–1.2 nm cut-off was applied for Van Der Waals and short-range electrostatic interactions.

**Fig. 6:**
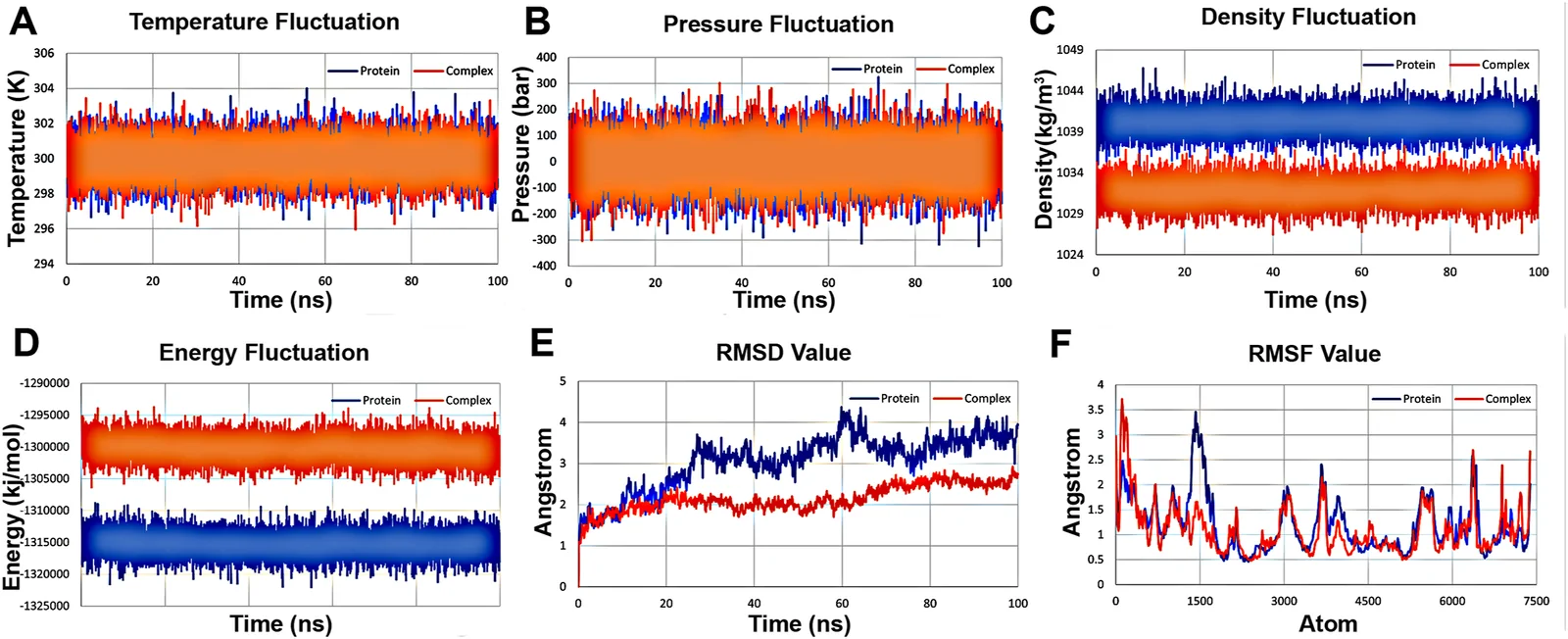
Molecular dynamics Simulations plot the 3EQM protein (blue) and SF04-3EQM complex (orange). (A) Temperature fluctuations of the protein and complex over time, (B) Pressure fluctuations over time, (C) Comparison of density change trends, (D) Energy stability analysis over time, (E) RMSD values reflect structural stability, and (F) RMSF values show differences in atomic flexibility. (For interpretation of the references to color in this figure legend, the reader is referred to the Web version of this article.)

#### 3.2.10 Root mean square deviation, and root mean square fluctuation

The RMSD trajectory of the apo protein and its FMN complex also increased to ∼4–4.5 Å after 20 ns, probably reflecting some observable (but not unusual) backbone flexibility in a cytochrome P450 enzyme, given that loop movement has been reported near the active site. The 3EQM–SF04 complex, however, did not exhibit such large fluctuations (2.0–3.0 Å), indicating that some backbone dynamics were suppressed by ligand binding. Notice that RMSD values higher than 3 Å are not ‘high stability’ in a strict sense, but moderate structural flexibility. By comparison, published MD studies of CYP19A1 and other CYP450 isoforms reveal RMSD fluctuations of 2–4.5 Å for stable complexes, depending on loop dynamics and the size of the bound ligand. Thus, although SF04 provides relative stabilization of 3EQM relative to the apo form, an expected conformational change still occurs in this system. The reduced fluctuations in the complex indicate greater structural stability and strong interactions between the ligand and the protein, as shown in Fig. 6.

It is an essential parameter in MDs to evaluate the structural flexibility of both the unbound protein and the protein-ligand complex. In the initial region, up to 1000 atoms, the complex exhibits a slightly lower RMSF compared to the apo protein, which stabilizes that region. In the middle area, a sharp peak is observed up to 3 Å in the unbound state of the protein, indicating a highly mobile segment. Later, it becomes rigid and stable upon binding with the ligand in the complex. In the range of 1500–5000 atoms, moderate fluctuations are observed in both the apo protein and the complex, indicating structural stability. In the last region of 6000–7500 atoms, the complex reflects ligand-induced stabilization with a lower RMSF value and minor fluctuations at the C-terminal due to conformational adjustment after binding, as shown in Fig. 6F.

#### 3.2.11 Radius of gyration, hydrogen bonds, SASA, and MMPBSA analysis

It represents the packing of protein atoms around their respective central mass. The stable, lower Rg values for the complex during the 100 ns simulation indicate an enhancement of protein compactness upon ligand binding. The unbound protein exhibits larger fluctuations, spanning a broader range, which means lower stability. In contrast, the ligand binding iindicates the protein's compactness and stability throughout the simulation, as shown in Fig. 7A. Hydrogen bonds play a crucial role in stabilizing the ligand-protein complex. Throughout the entire MD process, the presence of continuous hydrogen bonds maintained the dynamic equilibrium and stabilized the interaction between SF04 and the 3EQM. Initially, the complex forms 3–5 hydrogen bonds, which stabilize to 1–2 persistent hydrogen bonds throughout the trajectory, as shown in Fig. 7B. It maintains equilibrium by fluctuating between 213 and 225 nm^2^, exposing the protein surface to the solvent, suggesting stability and no conformational collapse. At the same time, the complex initially shows a higher Solvent accessible surface area **(**SASA) (215–228 nm^2^) during the first 20ns, but later on the fluctuations drop and stabilize the complex within 210–220 nm^2^ for the rest of the simulation, as shown in Fig. 7C. The molecular mechanics Poisson-Boltzmann Surface area approach was applied to estimate the total binding energy of the complex, providing an overall measure of its stability (SF04-3EQM) and the strength of interactions over a 100 ns simulation [36]. In this study, residue-wise decomposition was not performed; instead, different frames were extracted at regular intervals, and the total binding energy (ΔG) was computed and calculated for each segment to ensure the complex's stability throughout the simulation procedure. equation (1) of Gibbs Free energy of binding is [37]:

(1) ΔG_binding_ = ΔG_complex_ – (G_protein_ + G_ligand_)

**Fig. 7:**
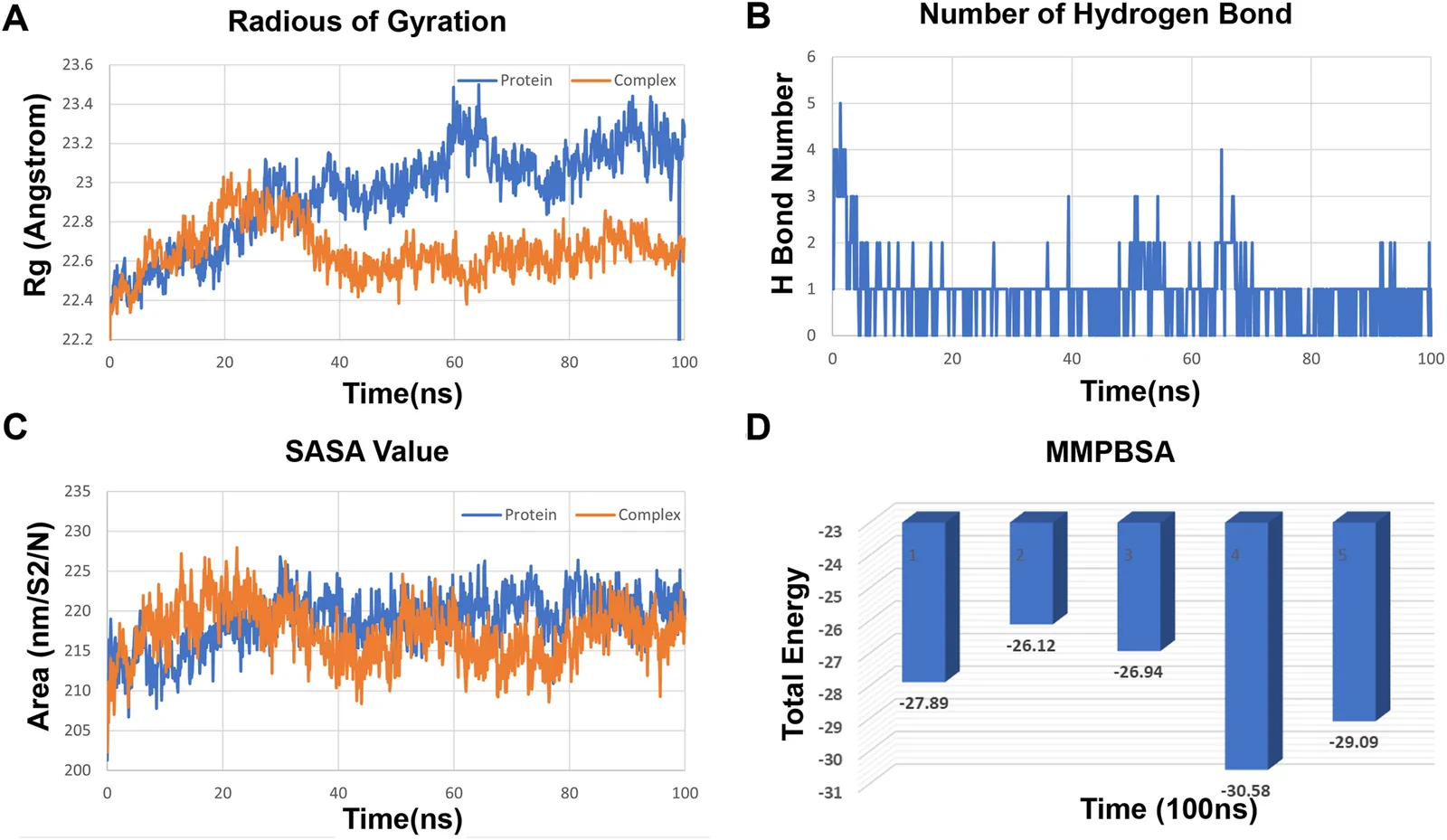
MD's profile showing 3EQM(blue), SF04-3EQM (orange). (A) Showing the trend of the radius of gyration of the protein and complex, (B) Analyzing the fluctuation of the number of hydrogen bonds in the system over time, (C) Comparing the SASA surface area changes of the protein and complex, and (D) MMPBSA results showing the change in the binding free energy of the complex. (For interpretation of the references to color in this figure legend, the reader is referred to the Web version of this article.)

The calculated binding energies ranged from −26.12 to −30.58 kcal/mol, with the most favorable energy value observed in the later stage of simulation. The overall average remained within the −26 to −30 kcal/mol range, indicating a strong, spontaneous binding affinity between SF04 and 3EQM. The negative free energies confirm the thermodynamic stability of complex formation.

The protein-ligand complex (3EQM-SF04) showed conformational adjustments at 0, 25, 50, 75 and 100 ns. At zero nanoseconds, the complex exhibited an initial stable conformation with compact secondary structure. At 25 ns, adjustment of the loops takes place with a slight relaxation, but the ligand (SF04) remains bound. At 50 ns, minor fluctuations are observed, indicating conformational adaptations (Fig. 8). Over time, at 75 ns, the structure achieved stability by maintaining the overall integrity and adjusting the fold in the complex. At 100 ns, the overall complex attained stability, confirming binding and structural equilibrium.

**Fig. 8:**
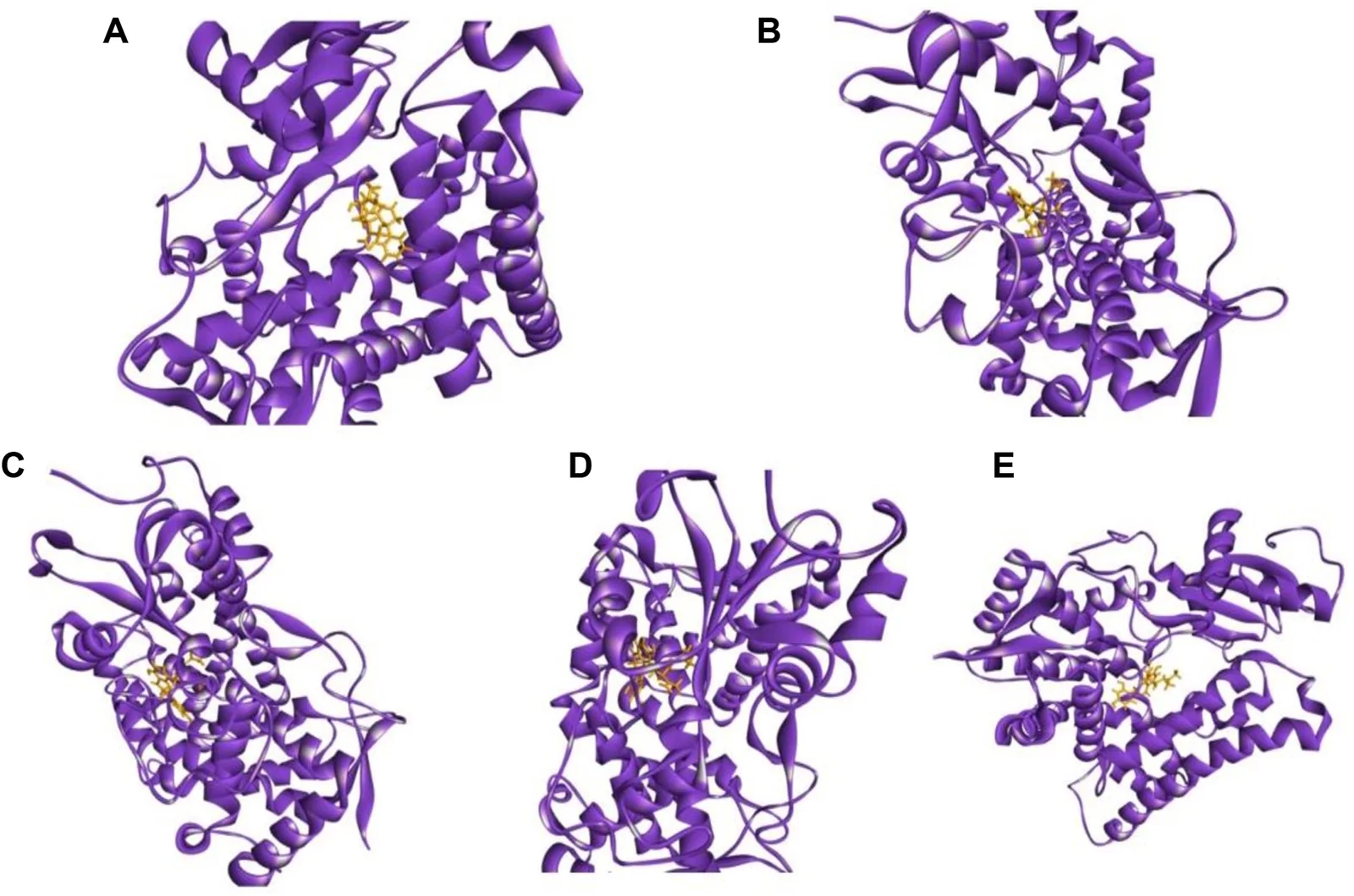
Show the molecular docking conformations of different complexes, with purple representing protein structures and yellow representing ligand molecules. Each figure shows the binding mode and spatial orientation of the ligand at the active site. Conformational adjustments of complex (3EQM-SF04) at (A) zero ns, (B) 25ns, (C) 50ns, (D) 75ns, and (E) 100ns.

#### 3.2.12 Density functional theory (DFT)

As Vogelin E (SF04) has shown promising results as an aromatase inhibitor in previous analyses, it was subjected to DFT calculations to evaluate its electronic properties for further validation. It is essential to discuss DFT parameters of the desired compound to understand the intermolecular interactions that reflect drug-like properties [38]. Different parameters, such as HOMO and LUMO calculations, the difference in chemical potential, Ionization potential, Electron affinity, Electronegativity, softness, hardness, and Electrophilicity index, are discussed here [39]. The SF04 structure was optimized, and DFT calculations were carried out. HOMO and LUMO calculations and the energy difference (ΔE = L-H) are shown in Table 5. Table 5 presents all the DFT parameters for SF04. The frontier molecular orbital values provide a sense of how SF04 might interact in the aromatase active site. The HOMO orbital distribution predicts sites in the molecule that can donate electron density, which is essential for hydrogen and π-π interactions with aromatic residues, including Phe148, Trp224, and Arg115. Similarly, the LUMO density indicates regions that can accept electron density from another molecule, thereby affecting its potential to engage in polar or charge-transfer interactions. The small HOMO-LUMO energy gap (ΔE) indicates a moderate chemical softness of SF04, which facilitates the flexible electronic polarization upon entrance into the heterogeneous electrostatic interior of the aromatase active site. This is a signature of ligands that can establish favorable complementarity through induced-fit interactions.

**Table 5:** Quantum chemical parameters of SF04 (Vogelin E).

Sr. #	Parameters	Calculation
1	HOMO (eV)	−0.20668
2	LUMO (ev)	−0.055
3	ΔE (eV)	0.015168
4	Ionization Potential (IP)	0.20668
5	Electron Affinity (EA)	0.055
6	Electronegativity (ꭕ)	0.13084
7	Chemical Potential (μ) (eV)	−0.13084
8	Hardness (η) (eV)	0.07584
9	Softness (S) (eV^−1^)	13.1856
10	Electrophilicity index (ω)	0.11286

#### 3.2.13 Frontier molecular orbital and molecular electrostatic potential

FMO related to HOMO and LUMO by describing interaction and stability analyses (Fig. 9). HOMO corresponds to electron donation, and LUMO to electron acceptance; the gap between HOMO and LUMO is known as the energy gap. The smaller the energy gap, the higher the stability and reactivity of the compound or molecule. The lower hardness and higher softness values of SF04 indicate greater ligand reactivity at the aromatase active site, thereby controlling different biological pathways, including oxidative stress and mitochondrial function. The value of electrophilicity indicates that nucleophilic centers, such as proteins and DNA, are prepared to interact with electrophilic ligands. The molecular electrostatic potential map describes the charge distribution over the surface. From a biological standpoint, the electrostatic potential map also rationalizes how SF04 might interact with key residues within the CYP19A1 active site. Electron-rich (red/yellow) regions might be potential hydrogen-bond acceptors that could form H-bonds with Arg115 or Met303, whereas electron-poor (blue) areas might represent potential hydrogen-donating groups oriented at residues like Ser199 or Ala307. The location of these zones is in good agreement with the hydrogen-bonding pattern observed during docking and MD simulation. Significantly, the intermediate electrophilicity index (ω) and the chemical softness (S) support the proposition that SF04 stabilizes via dispersive, π, and stacking interactions, which account for ligand binding in several CYP450 systems, including aromatase. The blue region represents electron-deficient areas favorable for nucleophilic attack, while the light green and yellow regions indicate intermediate potential values, corresponding to neutral zones [40]. Overall, the charge distribution across the molecule reveals its polarity and reactivity, which play a crucial role in its interactions with biological targets.

**Fig. 9:**
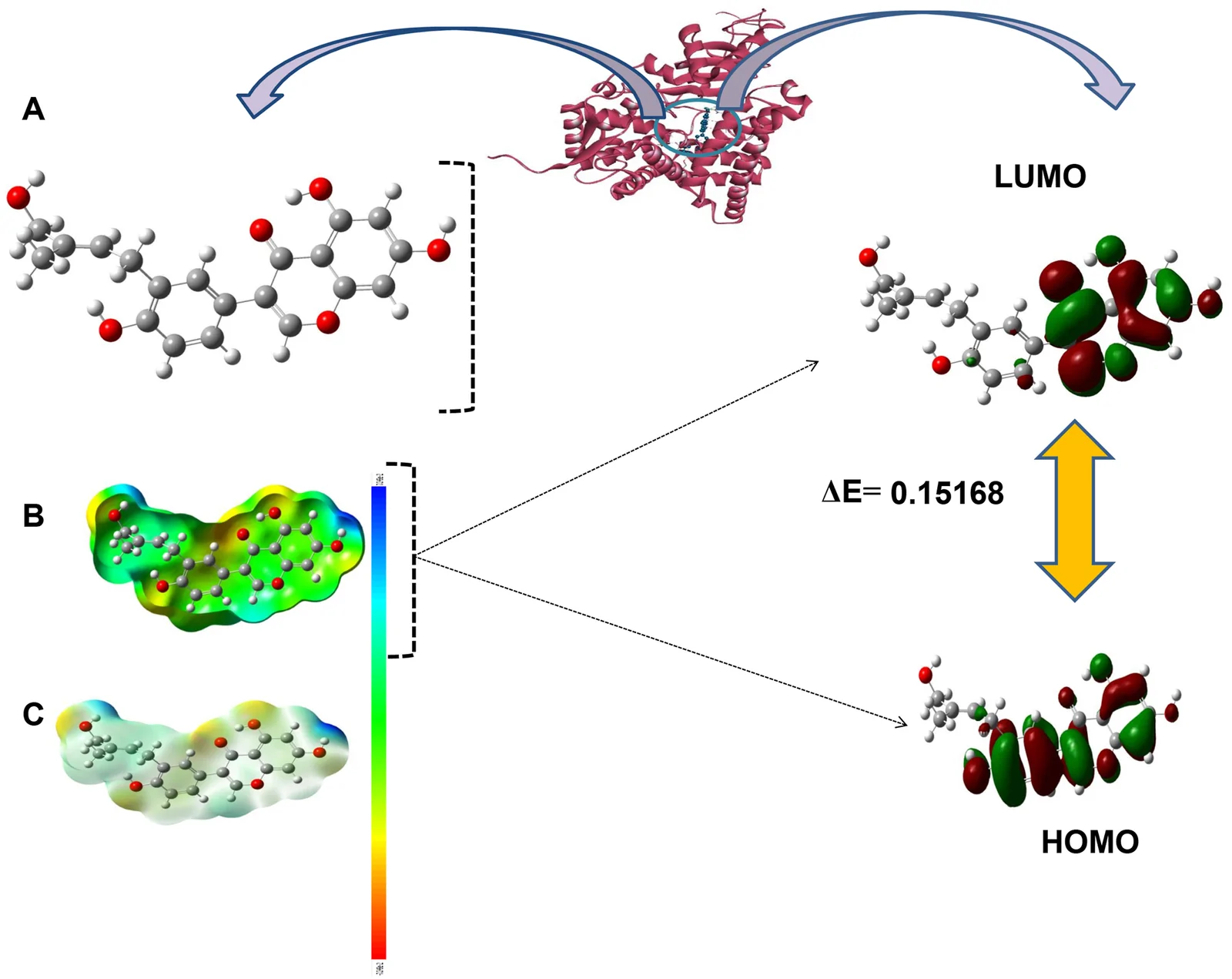
The DFT study of the stable molecule (SF04) embedded in 3EQM. (A) Three-dimensional structure of the compound, (B) Electrostatic potential distribution diagram, showing the electron density region, and (C) Surface charge distribution of the molecule; The right side shows the frontier orbital energy level diagram of the molecule, displaying the distribution of the highest occupied orbital (HOMO) and lowest vacant orbital (LUMO) and their band gap ΔE = 0.15168 eV.

## 4 Limitations

This study is entirely computational research; it employed a multitude of in silico strategies (validated docking protocol, ADMET screening, Molecular dynamics studies, Network pharmacological intervention, and DFT analysis) to establish biological relevance, paving the way for further experimental investigations. This integrated in silico pipeline is generally recognized as a reliable early screening method for identifying drugs that are feasible to experiment with. Nevertheless, all the aforementioned computations were simulated with a controlled setup. Biochemical means of demonstrating biological effects could include enzyme inhibition, pharmacokinetic studies, and cell viability assays, whereas, for systematic evaluation, experimental strategies would be necessary. These data provide a strong preclinical rationale and underscore the importance of future laboratory validation to advance these computational findings into clinical treatment. As docking scores are only estimates of binding preference and not a quantification of absolute affinity, they should not be used to suggest better energies than those of clinically approved inhibitors. It would be highly beneficial to integrate experimental data (Ki/IC_50_) or state-of-the-art computational methods, such as free-energy perturbation (FEP) or residue-level MM-GBSA decomposition, which would most likely significantly strengthen the conclusions of the present study. There is a discrepancy between the grid box values used and the coordinates reported for the grid center, and the manuscript provides little explanation of how particular active-site coordinates were selected for docking.

## 5 Conclusion

SF04 (Vogelin E) is a potent aromatase inhibitor with strong binding affinity and favorable pharmacokinetic properties, while exhibiting low toxicity, making it a promising candidate for drug design. Network pharmacology studies have shown that this compound is associated with multiple signaling pathways regulating estrogen synthesis, which play a crucial role in hormone-dependent breast cancer in postmenopausal women. Molecular dynamics simulations further validated the stability and potential therapeutic value of the SF04-target protein complex. Density functional theory (DFT) analysis also demonstrated the compound's high chemical stability and reactivity from an electronic structure perspective, providing a theoretical basis for drug design. These results collectively indicate that SF04 (Vogelin E) has a significant inhibitory effect on aromatase and possesses the potential to become a safe and effective candidate drug for breast cancer treatment. This study is entirely dependent on in silico models, and the computational/biochemical work lacks experimental validation, such as in vitro binding assays, aromatase inhibition assays, or cellular studies to support the in silico observations.

Furthermore, this study highlights the importance of utilizing naturally derived compounds as anticancer drugs. Compared with existing FDA-approved medications, such as letrozole and anastrozole, Vogelin E not only exhibits superior binding affinity and safety but also fewer adverse reactions and greater drug compatibility. Future research should focus on the in vitro and in vivo validation of this compound and its derivatives to evaluate their anticancer activity in different breast cancer cell lines. In further work, we need to validate the in vitro and in vivo anticancer activity of this compound and its derivatives in other breast cancer cell lines. Simultaneously, structural optimization and targeted modification can further enhance its bioavailability and selectivity, providing new directions and theoretical foundations for the development of novel aromatase inhibitors based on natural products. This work offers new research ideas and potential clinical applications for targeted therapy of breast cancer. The MD simulations indicate that SF04 binds in the aromatase pocket with moderate stability, which is not far from typical CYP450-ligand interactions and does not reflect unusually strong or more rigid binding. Although SF04 (Vogelin E) was the better-predicted binder and preferred computational safety profile compared with standard inhibitors, these were only predictive comparisons. In silico methods cannot predict true inhibitory potency, pharmacodynamics, toxicity, or clinical activity. Thus, SF04 should be considered a promising lead compound, and its predicted activity should first be confirmed by in vitro/in vivo experimental studies before a direct comparison with FDA-approved aromatase inhibitors.

## CRediT authorship contribution statement

**Laiba Rashid:** Writing – original draft, Software, Methodology, Investigation, Formal analysis, Conceptualization. **Jarin Tasnim:** Writing – original draft, Visualization, Software, Resources, Investigation, Formal analysis, Data curation, Conceptualization. **Md Mehedi Hasan:** Writing – original draft, Software, Resources, Investigation, Formal analysis. **Hammad Ismail:** Writing – review & editing, Software, Resources, Investigation, Formal analysis, Data curation. **Khan Rajib Hossain:** Writing – review & editing, Visualization, Software, Resources. **Sarnop Sarker:** Writing – review & editing, Software, Resources. **Ayesha Younas:** Writing – review & editing, Resources. **Muhammad Mujtaba:** Writing – review & editing, Software, Resources. **Mohammad Taufiq Alam:** Writing – review & editing, Supervision, Conceptualization.

## Declaration of competing interest

The content of the article is free of pertinent conflicts of interest and is not sponsored by any manufacturer or associated business, either directly or indirectly. There are not any confidentiality leaks, disagreements over other people's projects or patented technologies, duplicate submissions of the same manuscript, or authorship disputes in the content.

## Ethics approval

Not applicable.

## Declaration of generative AI in scientific writing

No generative AI tools have been used throughout the entire writing process of this manuscript.

## Funding information

There was no agency funding used in the drafting of this manuscript.

## Declaration of interest statement

The authors declare that they have no known competing financial interests or personal relationships that could have appeared to influence the work reported in this paper. All authors have read and approved the final version of the manuscript and agree with its submission to the journal.

## Data Availability

All data generated or analyzed during this study are included in this published article. Additional datasets or simulation files are available from the corresponding author upon reasonable request.
