# Corrigendum to “Computational screening of *Sophora flavescens* phytochemicals as potential aromatase inhibitors: Molecular docking, ADMET, Network pharmacology, Molecular dynamics simulation, and DFT studies” [Pharmaceutic. Sci. Adv. 4 (2026) 100108]

**DOI:** 10.1016/j.pscia.2026.100150

**Published:** 2026-09-11

**Authors:** Laiba Rashid, Jarin Tasnim, Md Mehedi Hasan, Hammad Ismail, Khan Rajib Hossain, Sarnop Sarker, Ayesha Younas, Muhammad Mujtaba, Mohammad Taufiq Alam

**Affiliations:** a School of Chemistry, University of the Punjab, Lahore, Punjab, 54590, Pakistan; b Department of Applied Chemistry and Chemical Engineering, University of Rajshahi, Rajshahi, 6205, Bangladesh; c Department of Biochemistry and Biotechnology, University of Gujrat, Punjab, 50700, Pakistan; d Department of Natural Science, BGMEA University of Fashion and Technology, Dhaka, 1230, Bangladesh; e Materials Engineering, University of Chinese Academy of Sciences, Beijing, 100040, China

The authors regret:

1. Reference 1 was cited in the Introduction to support a statement about breast cancer epidemiology. However, the cited article (“Investigation of wound healing and anti-inflammatory activity of *Senna occidentalis* leaf extract”) is irrelevant to the context of breast cancer and aromatase biology.

The reference has been removed and replaced with a more appropriate citation that is directly relevant to the subject matter, which has been listed below:

Maiuthed, A., Chatwichien, J., Sandech, N., Yang, M. C., Krobthong, S., Rukthong, P., Jisnuson S, Somsak R, Eurtivong, C. (2025). An integrated computational, in vitro and metabolomics approach to the discovery of novel aromatase inhibitors with anti-breast cancer activities. Scientific Reports, 15(1), 40230. https://doi.org/10.1038/s41598-025-24086-5.

2. In Section 2.2 (Molecular docking), there was a discrepancy between the stated grid box values and the coordinates reported for the grid center. The values were internally inconsistent, raising questions about the reproducibility of the docking protocol.

The stated grid center coordinates have been revised to align with the grid box values used in the actual docking experiment, which should be:

The grid box was positioned at the active site (heme bound by CastP), with coordinates of x = 85.370, y = 50.330, z = 41.570, and it centers at x = 20 Å, y = 20 Å, z = 20 Å, to determine the accurate placement of ligands.

3. There was a ten-fold numerical discrepancy for the HOMO-LUMO energy gap (ΔE) of SF04 (Vogelin E). Table 5 reported a value of 0.015168 eV, while the caption for Fig. 9 reported a value of 0.15168 eV.

The incorrect value of 0.015168 eV in Table 5 and its previous appearance in Fig. 9 caption has been corrected. Both now consistently report the energy gap as 0.15168 a. u. (atomic units) in the table, and the figure caption has been updated to reflect the corresponding value of 0.15168 a. u. to ensure consistency with the calculated data and units.

4. The original manuscript emphasized the identification of Vogelin E (SF04) as a “potent aromatase inhibitor.” As a fully computational (*in silico*) study, it is important to reiterate that these findings are predictive and lack direct experimental validation.

The conclusion has been tempered to state that SF04 is a “promising lead compound” or “potential candidate” rather than a confirmed “potent inhibitor.” The revised text now explicitly states:

Furthermore, this study is entirely based on computational (*in silico*) methods, and all results are derived from theoretical predictions, lacking in vitro and in vivo experimental validation. Therefore, the current results only indicate that SF04 is a potential aromatase inhibitor candidate compound, but cannot directly prove its actual inhibitory activity, pharmacodynamic properties, or clinical therapeutic effects.

The authors would like to apologise for any inconvenience caused.

